# Radiologic imaging and its current importance in breast cancer management

**DOI:** 10.3389/fradi.2025.1689635

**Published:** 2026-01-27

**Authors:** Nisha Dagar, Monu Sarin, Piyush Yadav, Rajdeep Thidwar

**Affiliations:** 1 Department of Radiology, Centre of Allied Health Sciences/Amrita School of Medicine, Amrita Institute of Medical Sciences and Research Centre, Faridabad, India; 2 Department of Radio-Diagnosis, SGT University, Budhera, Gurugram, India; 3 Guru Nanak Paramedical College, Dhahan Kaleran (PB), India

**Keywords:** cancer, CECT, magnetic resonance mammography, breast, mammography, contrast

## Abstract

**Introduction:**

This study examines the approaches used for cancer screening in women, insisting on the roles of mammography, breast ultrasound, breast MRI, computed tomography (CT), and positron emission tomography. Mammography is still the key method for early detection, with modern digital technology boosting image quality and diagnostic accuracy. Breast MRI provides increased sensitivity for high-risk individuals, and it is recommended to be used with mammography for full screening.

**Methods:**

This review article provides a comprehensive assessment of breast imaging techniques, focussing on screening recommendations and advancements in mammography, ultrasonography, MRI, CT, and PET. These methods provide a multidisciplinary approach to improving breast cancer detection, diagnosis, and personalized care. Early detection is necessary for less aggressive treatment. Digital breast tomosynthesis consists of contrast-enhanced spectral mammography, molecular breast imaging, MRI, and USG.

**Results:**

Mammography remains the primary imaging modality for detecting breast abnormalities, including cancer. The use of standardized interpretation systems improves the communication of findings. The present paper summarizes the primary disputes surrounding breast cancer management.

**Conclusion:**

According to the literature, low-dose mammography is the only radiographic technique that has significantly impacted the diagnosis, staging, and patient follow-up for asymptomatic breast cancer screening. Mammography is also the only accurate screening test for breast imaging. Implications for practice.

## Introduction

Worldwide, the cancer of breasts is the most common to strike women. According to studies, almost 2 million women are diagnosed yearly (1). The disease's heterogeneity, with multiple subtypes, adds to its complexity; however, early detection programs combined with advances in staging and imaging techniques have improved Rates of recovery for breast cancer patients by providing better options for treatment and planning than when surgery was the mainstay of care (2). Breast cancer screening lowers breast cancer-related mortality, and early discovery allows for less aggressive treatment. However, screening procedures are far from optimal due to large false-positive rates and poor sensitivity (3). Breast lumps can have a variety of causes, ranging from benign to malignant. The most common benign breast lump is fibroadenoma, and the most common cancer is invasive ductal carcinoma. A precise and efficient evaluation can boost cancer detection while reducing unnecessary tests and procedures (4). Mammography, particularly screening mammography, is the most effective tool for early breast cancer detection and is recommended once a year for women at average risk beginning at age 40. Digital mammography improves interpretation, particularly in patients with thick breast tissue (5). Radiologic imaging, such as ultrasonography, mammography, and MRI, is critical for early identification and diagnosis of breast cancer, resulting in much higher survival rates. It also permits imaging-guided interventions, which enhance the radiologist's multidisciplinary breast cancer management role (6). Radiologic imaging is essential in breast cancer management for correct staging, determining treatment response, and making therapeutic decisions. In metastatic breast cancer, CT, WB-DWI-MRI, and FDG-PET/CT improve disease assessment, altering treatment protocols and patient outcomes (7). Radiologic imaging is critical for detecting early breast cancer, staging it, and evaluating therapy options. PET-CT, BSGI, and DWI techniques improve detection accuracy, especially in dense breasts, allowing for more prompt and personalized therapeutic interventions and, ultimately, better patient outcomes. Each approach has its merits and limits; some are more effective in spotting small or thick tumours (8). Preoperative breast MRI is very important in breast cancer care because it gives a more reliable diagnosis of tumour size, greatly impacting surgical treatment decisions compared to mammography and ultrasound, which have greater rates of size misestimation (9). Radiologic imaging is critical in breast cancer management, especially in detecting metastatic lymph node involvement and guiding neoadjuvant systemic therapy, which has led to less extensive surgical methods and improved patient outcomes in recent decades. The significance of lymph node management in breast cancer, the function of neoadjuvant therapy, the evolution of surgical methods, and the advances in imaging technologies that are driving contemporary treatment approaches (10). Mixed imaging approaches can provide additional information to aid staging and therapeutic planning. The imaging goal was to develop a minimally intrusive therapy that would produce greater results while reducing adverse effects. The most essential component in reducing the mortality rate from some cancers is early detection by imaging-based screening. Carcinoma of the breast is the most often diagnosed malignancy among women. Breast imaging includes sonography, mammography, and scanning with magnetic resonance tomography (MRT) of the breast and is considered the second leading cause of cancer deaths in women. Further technological advancements will result in faster imaging speeds to match the demands of physiological processes. Particularly among the challenges in diagnosing breast cancer is sensibility. Complementary imaging examinations can help overcome this constraint utilized that traditionally include screening ultrasound, and combined mammography and ultrasound (11). Screening is critical in breast cancer management by assisting in early detection, correct diagnosis, treatment planning, and monitoring response to therapy, eventually improving patient outcomes and guiding interventional procedures. Radiological imaging aids in cancer diagnosis and staging, and it also plays a part in therapy planning (12). Mammography is currently the most common and available method for early detection of breast cancer. The most frequent breast abnormalities that can suggest breast cancer are lumps and calcifications. The goal is to promptly and accurately overcome the development of breast cancer, which is affecting an increasing number of women worldwide. Masses occur in a mammogram as small, granular clusters that are difficult to discern in a raw image. A mammography is one of the most effective breast cancer diagnostic methods available today. Breast cancer is discovered in its advanced stages using mammography images. Some simple segmentation techniques have been created to make a supporting tool for a simpler and less time-consuming way of identifying abnormal masses in mammography images (13).

## Methods used in this paper (screening recommendations)

### Role of mammography

Mammography is the primary imaging approach for detecting breast abnormalities, notably breast cancer. It is the only method recognized as a valid screening tool because of substantial study and technological advances. There are two primary forms of mammography: screening Mammography is used for routine examinations in women who do not have symptoms to detect potential problems early, while diagnostic mammography is used for people who do have symptoms or for follow-up after therapy to examine specific concerns. Digital systems use electronic detectors to generate images that can be viewed immediately. This method accelerates image processing and storage, enabling more efficient data analysis. High-quality pictures are required in mammography. Standardize how breast exams are read and reported. This technique divides findings into clear categories, allowing doctors to express the significance of the data and plan the next actions (14). An American study found a modest drop in mammography for screening in women aged 40–49 years after the publication of the USPSTF guidelines. It is noteworthy to note that the screening rate for this group increased during the next two years (15). Even while mammography screening has improved the detection of Ductal carcinoma *in situ* (DCIS) and initially invasive malignancies, there has been no significant shift in the prevalence of aggressive malignancy over the last thirty years. Data analysis from the National Cancer Institute's Surveillance, Epidemiology, and End Results (SEER) program, conducted between 1979 and 2008, indicated a shocking increase of 122 early breast cancers per 100,000 women. However, throughout the same period, the number of late-stage tumours reduced by 8% (16).

### Role of breast ultrasound

Women with dense fibroglandular tissue and low mammography detection rates are ideal candidates for breast screening ultrasonography. Dense breasts often have decreased mammography sensitivity, occasionally decreasing as low as 30%–48% (17, 18). In mammograms, dense breasts often contain at least 50% glandular tissue (ACR classifications 3 and 4).

The American College of Radiology (ACR) also advises breast ultrasonography in addition to mammography for women who are at high risk of breast cancer but cannot tolerate an MRI. Women who have had breast irradiation between the ages of 10 and 30, have a mutation in the BRCA gene, are related to a BRCA carrier, or have a lifetime risk of at least 20% for breast cancer are considered high risk (19). ACRIN 6666, a multicentre trial that investigated ultrasonography in women at extremely high risk of breast cancer, discovered that screening ultrasound might uncover 3.7 additional tumours per 1,000 screens in this cohort (20). Six studies were undertaken from 1995 to 2004 to evaluate screening ultrasonography in women with an average risk of breast cancer. There were 2,838 examinations in these six investigations, with only 126 patients having 150 more tumours discovered by breast ultrasonography. Ninety percent of the women had thick or heterogeneously dense parenchyma (21). Because of its high sensitivity and early detection rates—particularly for women with dense breasts—and its affordability in contexts where mammography is not available, breast ultrasonography (US) is a crucial tool for cancer screening, especially in low-resource settings (22). This brings validity to the idea that screening using breast ultrasonography is advantageous for women with thick breasts.

### Breast MRI

Additionally, there was not a randomized study to ascertain whether MRI lowers the overall fatality rate from breast cancer (23). In high-risk patients, the sensitivity of mammography screening in conjunction with MRI is higher (90%–100%) than mammography screening alone (25%–59%). However, the combined approach has a lower specificity (73%–93%) (24). For women with a high risk of breast cancer, the American Cancer Society (ACS) advised yearly screening MRI in 2007 to complement annual screening mammography, based on the findings of nine trials (25). In 2010, starting at age 30, the American College of Radiology and the Society of Breast Imaging advised yearly MRIs and mammograms for BRCA ½ carriers. For women who have a 20% or higher lifetime risk of breast cancer, a similar guideline is applicable. Women who claim have undergone prior chest radiation therapy should start yearly mammograms and MRI screenings at least 8 years after finishing treatment, but no earlier than age 25. From the moment of diagnosis, MRI and yearly mammograms should be taken into consideration for women with a history of breast cancer, biopsy-proven lobular neoplasia, or ovarian cancer (26).

### Role of CT in breast

Ultrasonography and mammography are augmented by the highly sensitive imaging technique, contrast-enhanced computed tomography (CE-CT). Researchers in medicine have assessed computed tomography's capacity to differentiate between benign and malignant tumours. However, contrast-enhanced computed tomography is sufficient for assessing the extent of tumour extension within the breast and for identifying lesions that are missed by other techniques because of its high spatial resolution and relatively low specificity.

Helical CT technology facilitates faster, gap-free scans and lowers x-ray doses when compared to traditional CT (27). Breast-conserving treatment (BCT), local therapy, or mastectomy are now the options available to women with early-stage breast cancer. It is crucial to perform a preoperative evaluation of tumour extension to choose candidates who qualify for BCT. This will involve multicentricity, a large intraductal component, and breast-daughter lesions (28). CT scans help detect pathological alterations suggestive of breast cancer by providing fine-grained images of the internal anatomy of the breast. Using a Convolutional Neural Network, this study classified breast cancer from CT scan pictures with an accuracy of 97.26% (29). With a sensitivity of 84.21%, specificity of 99.3%, and accuracy of 98.68%, chest CT scans are more sensitive than mammograms in the diagnosis of breast cancer. CT is a possible substitute for breast cancer screening since it may detect breast lumps and lymphadenopathy with accuracy (30).

### PET Imaging of Breast Cancer

As the use of molecular imaging for patients with breast cancer grows, breast radiologists must have a basic understanding of molecular imaging, especially PET. Current research on the FDA-approved PET tracer 16α-18F-fluoro-17β-estradiol (FES), which targets ER, includes guidelines examining the Association of Nuclear Medicine and Molecular Imaging on the appropriate use of FES-PET/CT for breast cancer and areas of active investigation for other potential applications (31). Primarily, the focus is on the utility of 18F-fluorodeoxyglucose (FDG) PET in staging, recurrence detection, and treatment response evaluation. Furthermore, there will be a delve into the growing interest in precision therapy and the development of novel radiopharmaceuticals targeting tumour biology. This includes discussing the potential of PET/MRI and artificial intelligence in breast cancer imaging, offering insights into improved diagnostic accuracy and personalized treatment approaches (32).

New digital technologies like Digital Breast Tomosynthesis (DBT) and Contrast-Enhanced Spectral Mammography (CESM) improve diagnostic capability over conventional mammography through:

- DBT (3D mammography): Reduces tissue overlap, enhancing lesion visibility, especially in dense breasts, and reduces false positives.
- CESM: Levitates use of contrast agents to accentuate tumor vascularity, improving detection of malignancies easily missed in conventional mammograms.
- SPR (Surface Plasmon Resonance)-based biosensing eliminates Imaging demerits through the Enabling label-free, real-time molecular sensing of biomarkers of cancer in fluids or blood, offering early high sensitivity and specificity, even prior to detecting a tumor on a picture, being non-invasive and potentially more cost-effective for screening and monitoring earlier.
- Thus, SPR biosensing complements imaging in offering earlier biochemical detection, avoiding the drawback of radiation exposure and reduced sensitivity in thick tissues.

## Result

Mammography is still the principal imaging modality for detecting breast abnormalities, including breast cancer. Screening mammography for asymptomatic women to discover early concerns and diagnostic mammography for symptomatic individuals or follow-up exams. Digital mammography improves image quality, processing, and storage, resulting in higher diagnostic accuracy. The use of standardized interpretation systems enhances the communication of findings. While mammography has greatly improved the diagnosis of early-stage malignancies, aggressive malignancy rates have remained stable over the last three decades. SEER program statistics from 1979 to 2008 show a 122% rise in early detections but only an 8% decrease in late-stage cases. Ultrasound complements mammography, especially in women with thick breasts, where mammographic sensitivity declines to 30%–48%. It is also indicated for high-risk women who cannot get an MRI. The ACRIN 6666 study found that ultrasound can detect an extra 3.7 tumours per 1,000 tests in high-risk patients. Its low cost and great sensitivity make it essential in low-resource environments and dense breast instances. MRI provides higher sensitivity (90%–100%) than mammography alone (25%–59%) for high-risk patients, but lower specificity. Annual MRI and mammography are indicated for women individuals who were with BRCA mutations who are at risk for breast cancer, beginning at age 30 or earlier based on specific risk factors. Contrast-enhanced computed tomography (CE-CT) is useful in determining tumour extent, especially for breast-conserving treatment (BCT). Helical CT is one example of an advancement that increases imaging speed while decreasing radiation exposure. CT's superior resolution allows for exact discrimination between benign and malignant tumours, with a reported sensitivity of 84.21% and specificity of 99.3%. AI applications, such as convolutional neural networks, have the potential for diagnosis accuracy better than 97%. PET-based molecular imaging is critical for staging, detecting recurrences, and assessing therapy outcomes. FDA-approved tracers, such as FES-PET/CT, target oestrogen receptors and offer data for precision medicine. Emerging areas include PET/MRI integration and the application of artificial intelligence to increase diagnosis accuracy and enable personalized treatment options.

## Conclusion

According to the literature, low-dose mammography is the only radiographic approach that has had a substantial influence on the diagnosis, staging, and patient follow-up for asymptomatic breast cancer screening. Mammography, or is the only precisely correct screening test for breast imaging. Though it is a useful screening tool, it has some limits, particularly in women with thick breasts. The latest evidence indicates that computer-aided detection, breast ultrasonography, and magnetic resonance imaging (MRI) are commonly used as adjuncts to mammography in modern clinical practice. These procedures improve the radiologist's ability to detect cancer and assess disease severity, which is essential for treatment planning and staging. Positron emission tomography, or PET, is also used to stage breast cancer and evaluate therapy outcomes. As imaging technology progresses, the role of imaging will change to reduce breast cancer morbidity and death. The progress in developing and commercializing the EIT breast imaging system will Support the dissemination of additional applications and infrastructure in line with EIT and similar depiction technologies. Currently, x-ray mammography is the most widely utilised breast-imaging technology, making it the “gold standard” in imaging-based breast examination.

Mammography is the standard for breast cancer screening because it is the only imaging modality to reduce mortality in large-scale studies, is highly prevalent, low cost, fairly quick, and sensitive to detection of early signs like microcalcifications. Other modalities like MRI, CT, and PET are less available, more costly, and better suited to diagnostic or high-risk conditions than population-wide screening. Digital Breast Tomosynthesis (3D mammography) improves detection in dense breasts, Contrast-Enhanced Mammography (CEM) offers functional imaging at no additional cost of MRI, Low-dose Molecular Imaging (e.g., PEM) offers high sensitivity without compression, AI-assisted screening improves accuracy, risk prediction, and workflow efficiency and Photoacoustic Imaging use ultrasound and light to offer functional imaging.

It would take a future replacement to be accurate, low in cost, scalable, and proven to reduce mortality.
